# Supplementary material for: Topography and Nanomechanics of the Tomato Brown Rugose Fruit Virus Suggest a Fragmentation-Driven Infection Mechanism
Source: Viruses. 2025 Aug 25;17(9):1160. doi: 10.3390/v17091160 (PMC12474120; doi:10.3390/v17091160)
Supplement: Supplementary file 1 [file viruses-17-01160-s001.zip › viruses-3796558-supplementary.pdf]

Supporting Information

Topography and nanomechanics of the tomato brown rugose fruit virus suggest a fragmentation-driven infection mechanism

Péter Puskás[1], Katalin Salánki[2], Levente Herényi[1], Tamás Hegedűs[1,3] and Miklós Kellermayer[1,3*]

[1]Department of Biophysics and Radiation Biology, Semmelweis University, Budapest Hungary

[2]Department of Plant Pathology, Plant Protection Institute, Centre for Agricultural Research, HUN-REN, H-1525 Budapest, P.O. Box 102., Hungary

[3]HUN-REN Biophysical Virology Research Group, Semmelweis University, Budapest Hungary

*Corresponding author: Miklós Kellermayer

Email: kellermayer.miklos@semmelweis.hu

Supporting Information Text

To test what effect force mapping had on the ToBRFV virions, we collected *a posteriori* AFM images (**Figure S5**). **Figure S5.a** shows the AC-mode height-contrast AFM image of the ToBRFV-coated mica surface prior to force mapping. **Figure S5.b** shows the same surface after acquiring the force map (**Figure S5.c**), and indicates that many of the virions have been dislocated or dislodged from the surface upon the nanomechanical experiment. The observation raises the possibility that the features of the force spectra (**Figure 3.d** of the main text) might be due to mechanically driven virion dissociation from the surface rather than to structural changes in the capsid itself. To test for this possibility, we re-scaled the topographical height color lookup table (**Figure S5.d**). The test revealed that the substrate surface contains granular material in exactly the same places where the ToBRFV particles were originally observed. We identify this granular material as the remnants of the ruptured capsid wall. Thus, the nanomechanical manipulation of individual ToBRFV particles indeed resulted in their force-driven rupture.

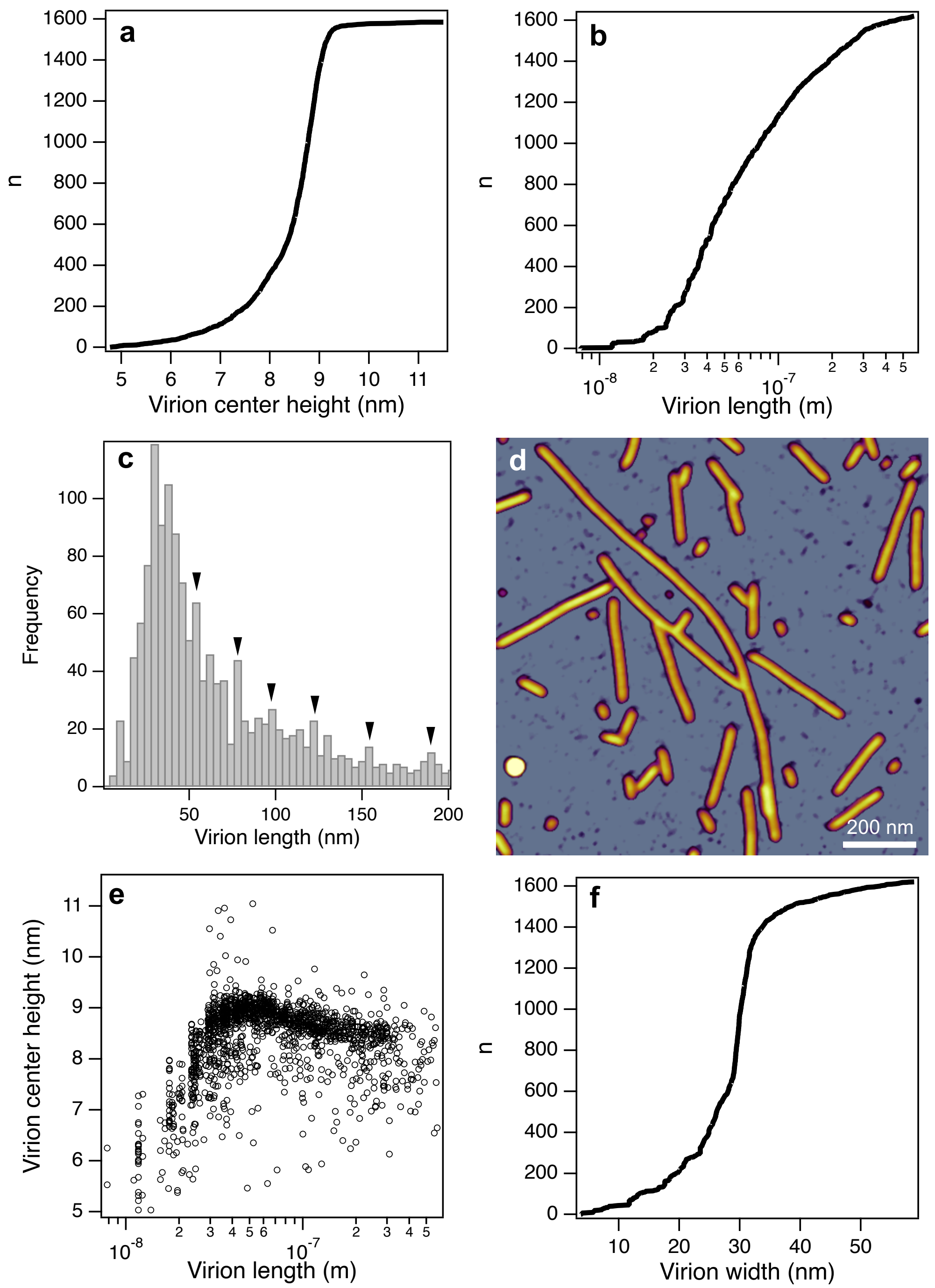

Figure S1. AFM imaging and particle analysis of ToBRFV. a. Cumulative distribution of virion center height. **b.** Cumulative distribution of virion length. **c.** Length histogram with linear axis scale, indicating local modes (black arrowheads). **d.** Example of an extremely long ToBRFV particle with a length of 1.33 μm. **e.** Virion center height as a function of length. **f.** Cumulative distributions of virion width.

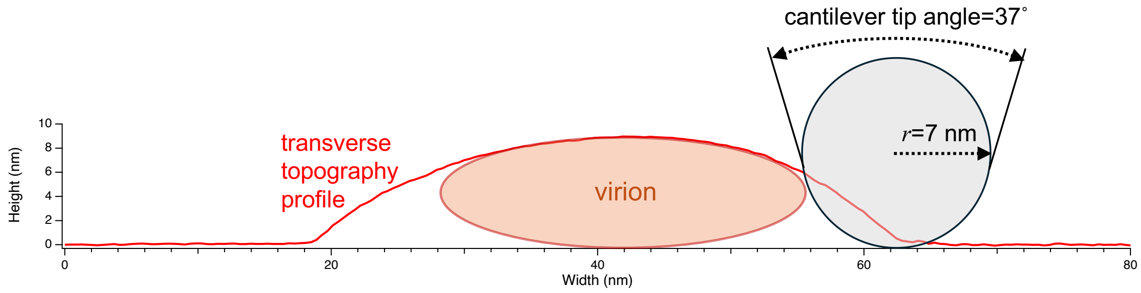

Figure S2. Scaling-corrected cross-section of the ToBRFV virion. Cross-sectional topographical profile plot of a ToBRFv particle with the same scaling along the x and y axes. The tip of the AFM cantilever (Olympus AC160TS) is shown to scale.

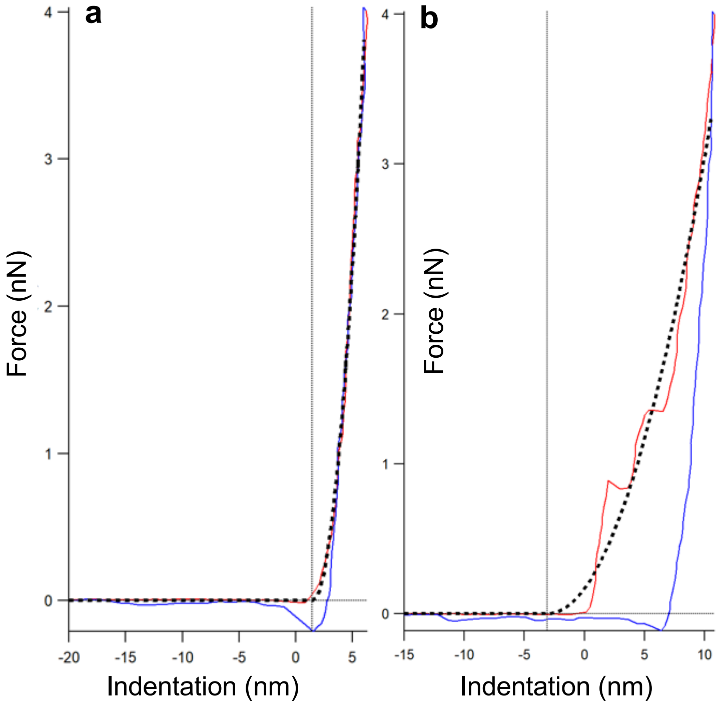

Figure S3. Force spectroscopy of ToBRFV. Examples of control (substrate surface, **a**) and ToBRFV virion (**b**) force curces with fits of the Hertz model (black dotted line).

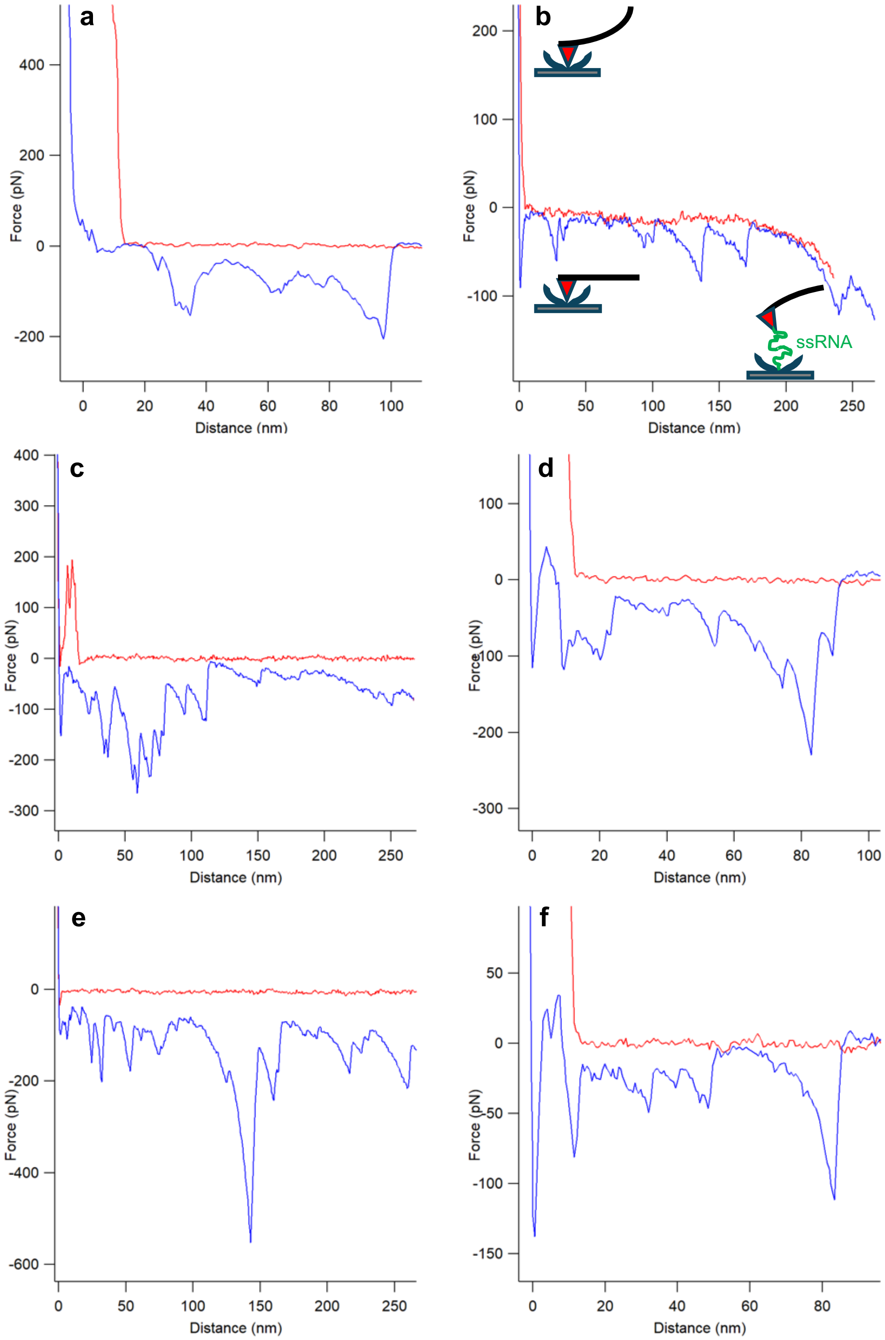

Figure S4. Mechanical manipulation of the ssRNA genome. Examples of force *versus* distance curves in which the retraction (blue) curves contained force peaks. The inset schematics in **b** provide interpretation of the nanomechanical data, indicating that the non-linear curves and force peaks that appear during cantilever retraction correspond to the extension and unfolding a long (>250 nm) polymer chain. The non-linear part of the indentation curve seen in **b** (red trace) corresponds to the relaxation of an ssRNA molecule unfolded and extended in the preceding mechanical cycle.

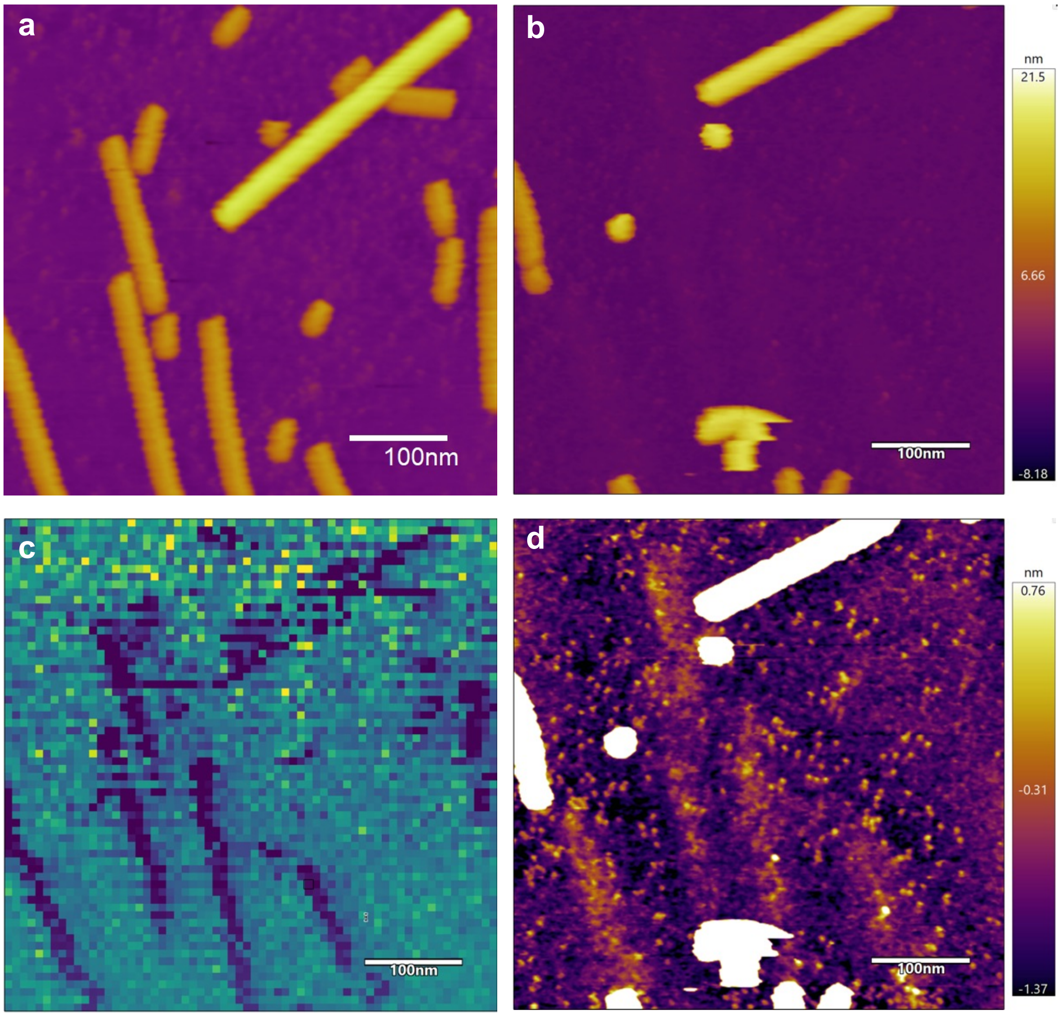

Figure S5. Force mapping of ToBRFV. Investigation of the substrate surface following force mapping. **a**. Height-contrast AFM image collected prior to force mapping (same as **Fig.4.a**). **b**. Height-contrast AFM image collected after force mapping. The image is shown with the same color lookup-table settings as in **a**. **c**. Force map, displaying the Young modulus disteibution across the sample surface (same as **Fig.4.b**). **d**. Height-contrast AFM image collected after force mapping (same as **Fig.6.b**). The image is shown with the same color lookup-table settings scaled to reveal small particles on the surface.

a MSYTIATPSQFVFLSSAWADPIELINLCTNSLGNQFQTQQARTTVQRQFSEVWKP
VPQVTVRFPDSGFKVYRYNAVLDPLVTALLGAFDTRNRIIEVENQANPTTAETLD
ATRRVDDATVAIRSAINNLAVELVKGTGLYNQSTFESASGLQWSSAPAS

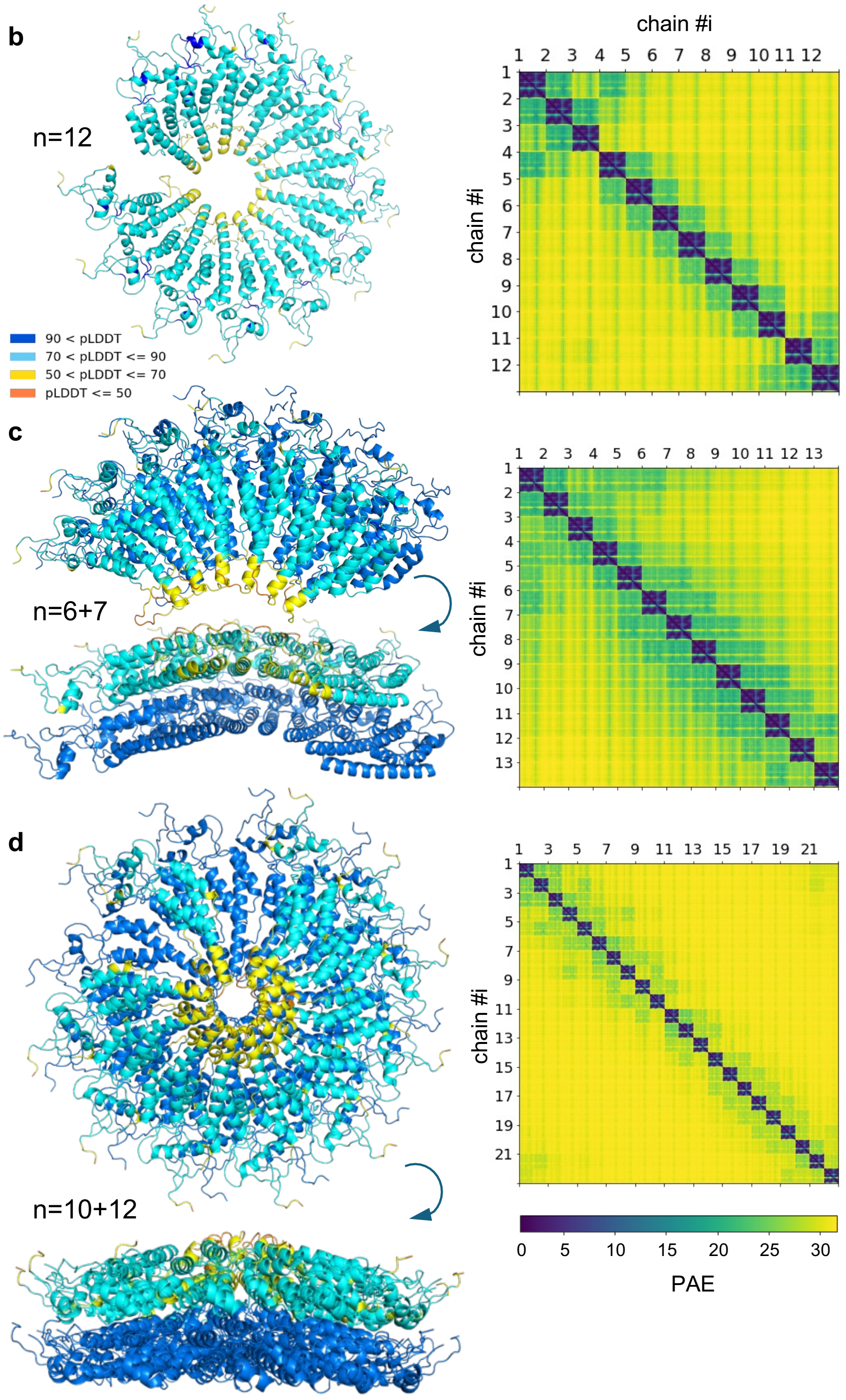

Figure S6. Prediction of ToBRFV coat-protein structure. a. Amino acid sequence of the ToBRFv 17.5 kDa coat protein (CP). **b–d.** Multimeric assemblies of ToBRFv were predicted using AlphaFold3. Structures are colored by pLDDT scores, combined with blue elements for visualizing

multilayered models. Predicted Alignment Error (PAE) plots are shown on the right, indicating the model's confidence in intra- and inter-chain interactions. **b.** With 12 chains, AlphaFold3 predicted a single disk-like structure featuring a gap, suggesting a potentially missing protomer. PAE values between protomers showed moderate to high errors (light green). **c.** With 13 chains, a partial disk spanning two layers was predicted. The PAE values were slightly higher and showed more consistency across protomer interfaces. **d.** With 22 chains, the model predicted nearly two complete disks arranged in two layers. However, the PAE values were high, and the inner diameter appeared small. This was the maximum number of protomers that could be predicted with our current hardware (GPU with 48 GB VRAM). Taken together, the 13-chain model, which forms a partial two-layered disk with relatively consistent confidence across protomers, appears to be the most reliable among our predictions and is also shown in **Figure 4.b** of the main text.

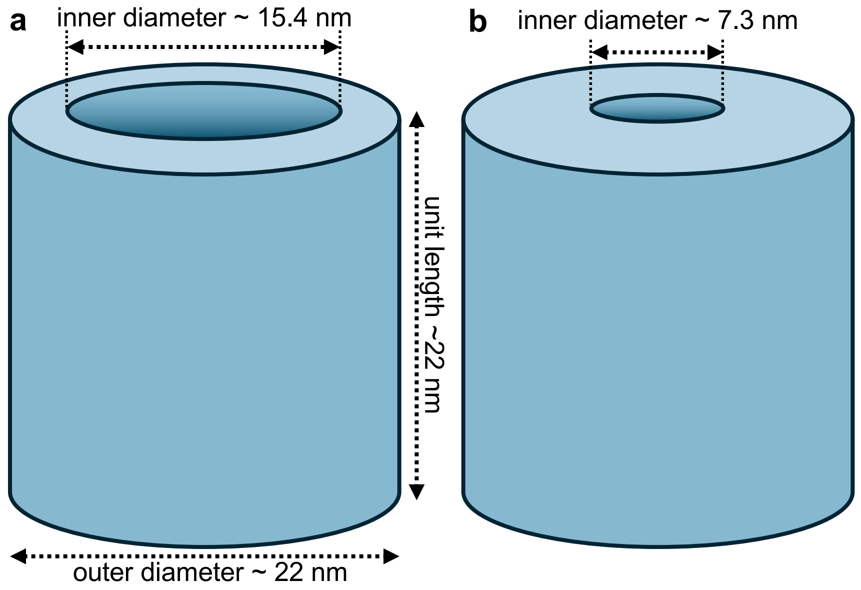

Figure S7. Schematic models of the ToBRFV structural unit. a. Model based on the estimation of the capsid wall thickness by nanomechanical experiments (see **Figure 3.h** of the main text). **b.** Model based on the estimation of coat-protein structure with AlphaFold (see **Figure 4** of the main text).
